# Heparanase Promotes Syndecan-1 Expression to Mediate Fibrillar Collagen and Mammographic Density in Human Breast Tissue Cultured *ex vivo*

**DOI:** 10.3389/fcell.2020.00599

**Published:** 2020-07-14

**Authors:** Xuan Huang, Gina Reye, Konstantin I. Momot, Tony Blick, Thomas Lloyd, Wayne D. Tilley, Theresa E. Hickey, Cameron E. Snell, Rachel K. Okolicsanyi, Larisa M. Haupt, Vito Ferro, Erik W. Thompson, Honor J. Hugo

**Affiliations:** ^1^Institute of Health and Biomedical Innovation, Queensland University of Technology, Kelvin Grove, QLD, Australia; ^2^Translational Research Institute, Woolloongabba, QLD, Australia; ^3^School of Biomedical Science, Queensland University of Technology, Brisbane, QLD, Australia; ^4^Faculty of Science and Engineering, Queensland University of Technology, Brisbane, QLD, Australia; ^5^Radiology Department, Princess Alexandra Hospital, Woolloongabba, QLD, Australia; ^6^Dame Roma Mitchell Cancer Research Laboratories, Adelaide Medical School, University of Adelaide, Adelaide, SA, Australia; ^7^Cancer Pathology Research Group, Mater Research Institute, The University of Queensland, Brisbane, QLD, Australia; ^8^Mater Pathology, Mater Hospital Brisbane, South Brisbane, QLD, Australia; ^9^Genomics Research Centre, School of Biomedical Sciences, Institute of Health and Biomedical Innovation, Queensland University of Technology, Kelvin Grove, QLD, Australia; ^10^School of Chemistry and Molecular Biosciences, The University of Queensland, Brisbane, QLD, Australia

**Keywords:** mammographic density, breast cancer, heparanase, syndecan-1, NMR

## Abstract

Mammographic density (MD) is a strong and independent factor for breast cancer (BC) risk and is increasingly associated with BC progression. We have previously shown in mice that high MD, which is characterized by the preponderance of a fibrous stroma, facilitates BC xenograft growth and metastasis. This stroma is rich in extracellular matrix (ECM) factors, including heparan sulfate proteoglycans (HSPGs), such as the BC-associated syndecan-1 (SDC1). These proteoglycans tether growth factors, which are released by heparanase (HPSE). MD is positively associated with estrogen exposure and, in cell models, estrogen has been implicated in the upregulation of HPSE, the activity of which promotes SDC expression. Herein we describe a novel measurement approach (single-sided NMR) using a patient-derived explant (PDE) model of normal human (female) mammary tissue cultured *ex vivo* to investigate the role(s) of HPSE and SDC1 on MD. Relative HSPG gene and protein analyses determined in patient-paired high vs. low MD tissues identified SDC1 and SDC4 as potential mediators of MD. Using the PDE model we demonstrate that HPSE promotes SDC1 rather than SDC4 expression and cleavage, leading to increased MD. In this model system, synstatin (SSTN), an SDC1 inhibitory peptide designed to decouple SDC1-ITGαvβ3 parallel collagen alignment, reduced the abundance of fibrillar collagen as assessed by picrosirius red viewed under polarized light, and reduced MD. Our results reveal a potential role for HPSE in maintaining MD via its direct regulation of SDC1, which in turn physically tethers collagen into aligned fibers characteristic of MD. We propose that inhibitors of HPSE and/or SDC1 may afford an opportunity to reduce MD in high BC risk individuals and reduce MD-associated BC progression in conjunction with established BC therapies.

## Introduction

In Australia, mammographic screening reduces mortality by around 50% for screening participants (Roder et al., 2008; Nickson et al., 2012). However, mammographic test sensitivity is impaired in women who have high mammographic density (HMD), where the dense area on the mammogram can mask BC-associated features, contributing to an increase in “interval cancers” that arise within 1–2 years of a “clear” mammogram, and an increase in false negative and false positive screens (Nelson et al., 2016). HMD is therefore an important impediment to effective screening.

After adjusting for age and body mass index (BMI), on a population basis HMD is also one of the strongest risk factors for BC. Breast cancer is currently diagnosed in 1 in 8 Australian women over their lifetime, and accounts for ∼19,500 cases and 3,000 deaths annually (AIHW, 2019), making BC the major cause of female cancer-associated death in Australia. HMD is common; ∼43% of women aged between 40 and 74 have heterogeneously or extremely dense breasts, wherein HMD regions represent greater than 50% of the total area (Sprague et al., 2014). An underappreciated fact is that HMD-associated BC risk is more impactful than other known risk factors, including the BRCA1/2 BC predisposition genes, when considered on a population-wide basis (Hopper, 2015). In addition to increased BC risk, evidence is also emerging that HMD is associated with increased BC recurrence (Hwang et al., 2007; Cil et al., 2009; Shawky et al., 2015, 2019; Huang et al., 2016) and treatment resistance (Elsamany et al., 2015). Thus, interventions that reduce MD may offer new avenues for the prevention, diagnosis and treatment of BC.

A standout molecular feature of HMD is the preponderance of extracellular matrix (ECM), largely comprised of collagen fibers and proteoglycans (PGs), which are carbohydrate-coated proteins (Huo et al., 2014; Shawky et al., 2015). Abnormalities in ECM have been implicated in many pathologies, including cancer (Lu et al., 2012). Importantly, targeting ECM components has proven efficacious for the treatment of some diseases (Jarvelainen et al., 2009; Ferro et al., 2012). In the breast, increased abundance and organization of collagen is associated with HMD (Boyd et al., 2010; Huo et al., 2015; McConnell et al., 2016) and our LMD vs HMD “within breast” comparative data have confirmed that collagen-rich ECM is the most discriminatory feature of HMD (Lin et al., 2011; Huo et al., 2015). Accumulation of collagen can influence mammary malignancy both *in vitro* (Provenzano et al., 2008; Levental et al., 2009) and *in vivo* (Provenzano et al., 2009), and the collagen profile of HMD predicts poor survival in BC (Conklin et al., 2011). Although dense collagen is undoubtedly a major feature of HMD-associated ECM, it forms a scaffold for a wide range of around 350 distinct ECM proteins, collectively called the “Matrisome,” which includes many PGs (Naba et al., 2012). HMD stroma has many similarities with BC-associated stroma, where both promote the progression of malignancy. To date, the specific components of HMD ECM that promote BC are unknown. One candidate of interest, SDC1, has been positively associated with both HMD and BC (Shawky et al., 2015). HMD is ∼60% genetically inherited (Boyd et al., 2002), and GWAS studies have identified single nucleotide polymorphisms (SNPs) in several PGs and in PG-modifying enzymes that correlate with increased BC risk (Shawky et al., 2015). In particular, we have identified SDC1 and SDC4 SNPs in BC (Okolicsanyi et al., 2015).

The heparan sulfate proteoglycan (HSPG) family of glycoproteins include membrane bound proteins SDC1 and SDC4, glypican (GPC 1-6), beta-glycan, neuropilin-1 and CD44 (hyaluronic acid receptor), secreted extracellular matrix components (agrin, perlecan, type XVIII collagen), and the secretory vesicle molecule, serglycin (Sarrazin et al., 2011). HSPGs are widely involved in biological activities, including cell signaling, cell adhesion, ECM assembly, and growth factor storage (reviewed in Nagarajan et al., 2018). HSPGs display primarily heparan sulfate-containing glycosaminoglycan (GAG) side chains that bind growth factors, which are in turn released by the action of HPSE (Shawky et al., 2015). This enzyme trims HS GAG chains from HSPGs (including SDC1), allowing the PG core proteins to be cleaved by enzymes such as MMP-9. For SDC1, this results in release from the cell membrane. Furthermore, a positive feedback loop occurs whereby SDC1 shedding stimulates its own expression (Ramani et al., 2012). Shed SDC1 is also taken up by BC cells to promote proliferation (Su et al., 2007), and has been shown to be important in Wnt signaling, contributing to tumorigenesis (Alexander et al., 2000).

HPSE expression strongly correlates with poor survival in BC (Sun et al., 2017) and serum levels of shed SDC1 have been identified to be informative in regards to progression of several cancers including breast (Joensuu et al., 2002; Vassilakopoulos et al., 2005; Szarvas et al., 2016). Shed SDC1 abundance detected in serum has been associated with BC size (Malek-Hosseini et al., 2017), and to be a result of chemotherapy (Ramani and Sanderson, 2014). SDC1 gene expression in BC can be prognostic (Cui et al., 2017), with its induction in stromal fibroblasts identified in invasive BC (Yang et al., 2011; Vlodavsky et al., 2012). Of relevance to BC, HPSE action to deglycanate SDC1 is essential for the binding of the core protein of SDC1 with lacritin, a protein known to be expressed in the breast, enabling mitogenic signaling (Weigelt et al., 2003; Ma et al., 2006).

Recent data identifying a positive association of HMD with lifetime exposure to estrogens is also a key finding to our understanding of MD in BC (Huo et al., 2015). Estrogen unequivocally promotes MD, as evidenced by data showing alterations of MD with hormone contraceptives, hormone replacement therapy (Greendale et al., 1999; Byrne et al., 2017), menopause (Stone et al., 2009), and treatment with anti-estrogenic drugs (Chew et al., 2014; Shawky et al., 2017). Estrogen also influences cells within the tumor microenvironment, causing immunosuppression (Rothenberger et al., 2018). However, the mechanistic basis of estrogen promotion of MD is, as yet, unknown. Crucially, however, estrogen can directly upregulate HPSE in MCF-7 cells (Elkin et al., 2003; Xu et al., 2007), and thus the E2/HPSE/SDC1 axis has strong potential as the mediator of MD effects on BC through autocrine paracrine pro-malignant actions.

We hypothesized that estrogen promotes HMD by up-regulating HPSE and SDC1 as part of its pro-oncogenic effects. Using anti-estrogens as well as HSPE inhibitors developed for cancer treatment and in use in clinical trials, and the SDC1 specific inhibitor SSTN, we investigated their effects in patient-derived explants of normal mammary tissue cultured over a 2 week period. Our data identified that MD change was detectable and linked to the estrogen-HPSE-SDC1 axis.

## Materials and Methods

### Patient Cohort

Human breast tissue specimens with no evidence of malignancy, and surplus to pathology needs, were accrued from prophylactic mastectomy surgeries, primarily in women with high BC risk, as determined by family history of breast cancer and/or with contralateral benign or malignant disease. Tissue from patients on recent (<6 months) hormone-based therapies or patients who carried BRCA1/2 mutations were excluded from the study. The demographics of the patients who donated their breast tissue for this study are detailed in Table 1. The study was approved by the Metro South Hospital and Health Services, Queensland (HREC/16/QPAH/107). Resected breast tissue was placed on ice and taken to the Pathology Department at the respective hospital, cut into 1–1.5 cm thick slices and checked for lesions. Up to 3 slices were then obtained and used in this project. Some tissues were processed for downstream analyses and some HMD regions were delegated to culture as patient-derived explants.

**TABLE 1:** Patient demographics from which tissue was examined for this study.

Patient ID	Age	Menopausal status	BIRADs density score
GPH008M	30	Pre	3
MPRIV015R	29	Pre	1
MPRIV022M	41	Pre	4
PAH001R	41	Pre	2
PAH005M	41	Pre	2
PAH010M	34	Pre	2
PAH021M	23	Pre	1
PAH025M	34	Pre	1
PAH033M	41	Pre	4
907	–	–	–
P15800	–	–	–
MATER004M	–	–	–
UR933036	48	Peri	2
GPH012M	53	Post	2
GPH019M	43	Post	2
PAH032M	48	Post	3
PAH040M	48	Post	4
			

### RNA Extraction and RT-qPCR

RNA was extracted from human breast tissue after homogenization into TRIzol (Ambion, Life Technologies) using Qiagen Tissue Lyser II with metal beads. At the 1:1 isopropanol: sample step, the solution was added to a Bioline RNA extraction column (Catalog no BIO-52075) to purify RNA according to manufacturer’s instructions. RT-qPCR was performed as previously described (Hugo et al., 2013). Primer sequences are detailed in Table 2.

**TABLE 2:** Sequences of RT-qPCR primers used in this study.

Gene	Sequence
L32 Fwd	GATCTTGATGCCCAACATTGGTTATG
L32 Rev	GCACTTCCAGCTCCTTGACG
SDC1 Fwd	CTGGGCTGGAATCAGGAATATTT
SDC1 Rev	CCCATTGGATTAAGTAGAGTTTTGC
SDC2 Fwd	AGCTGACAACATCTCGACCACTT
SDC2 Rev	GCGTCGTGGTTTCCACTTTT
SDC3 Fwd	CTTGGTCACACTGCTCATCTATCG
SDC3 Rev	GCATAGAACTCCTCCTGCTTGTC
SDC4 Fwd	CCACGTTTCTAGAGGCGTCACT
SDC4 Rev	CTGTCCAACAGATGGACATGCT
HPSE Fwd	TCACCATTGACGCCAACCT
HPSE Rev	CTTTGCAGAACCCAGGAGGAT
GPC1 Fwd	GGACATCACCAAGCCGGACAT
GPC1 Rev	GTCCACGTCGTTGCCGTTGT
GPC2 Fwd	TGATCAGCCCCAACAGAGAAA
GPC2 Rev	CCACTTCCAACTTCCTTCAAACC
GPC3 Fwd	GATACAGCCAAAAGGCAGCAA
GPC3 Rev	GCCCTTCATTTTCAGCTCATG
GPC4 Fwd	GGTGAACTCCCAGTACCACTTTACA
GPC4 Rev	GCTTCAGCTGCTCCGTATACTTG
GPC6 Fwd	CAGCCTGTGTTAAGCTGAGGTTT
GPC6 Rev	GATGTGTGTGCGTGGAGGTATGT
MMP2 Fwd	CTCCTGACATTGACCTTGGCA
MMP2 Rev	ATCAAGGGCATTCAGGAGCTC
MMP9 Fwd	GGACGGCAATGCTGATGGGAAA
MMP9 Rev	CGCCGCCACGAGGAACAAA
MMP14 Fwd	GCAAAGCTGATGCAGACACCATGAA
MMP14 Rev	CTCTCCCACACGCGGAAC
TFF-1 Fwd	GCAATGGCCACCATGGAGAACAA
TFF-1 Rev	GAGGGCGTGACACCAGGAAAA

### Immunohistochemistry

**FIGURE 1:**
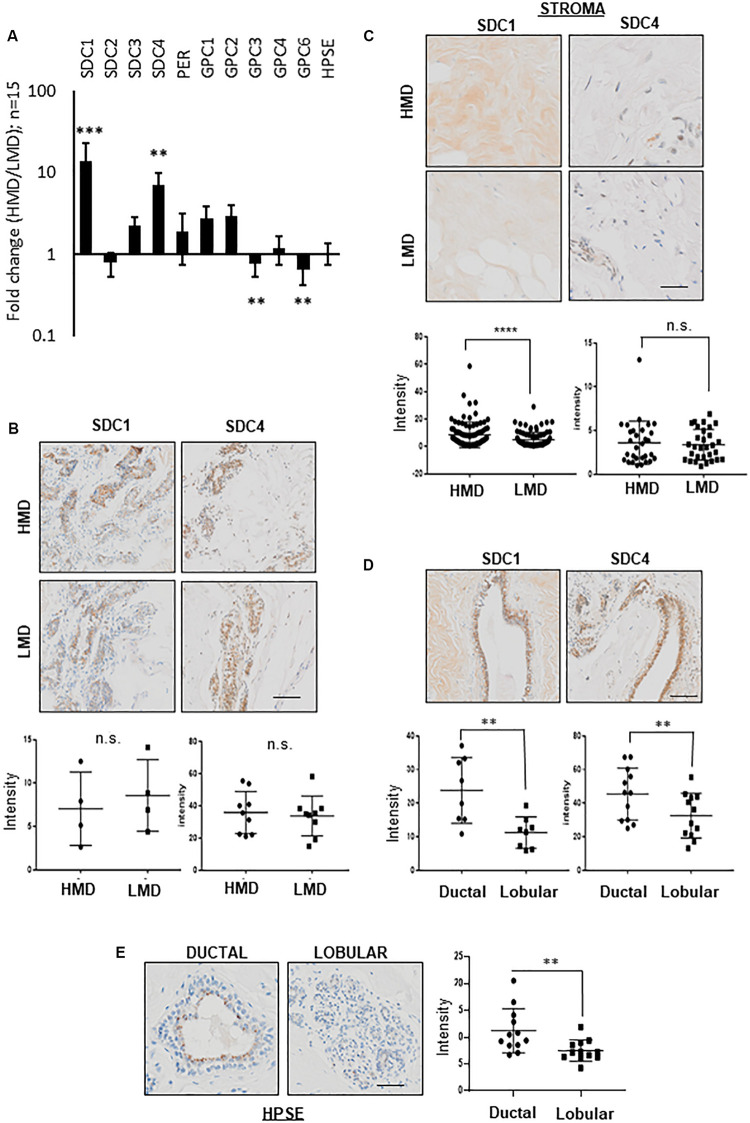
PGs expression in HMD vs. LMD. **(A)** RT-qPCR for various HSPG proteins and HPSE (HPSE) in HMD vs. LMD paired patient tissue. Fold change (HMD/LMD) gene expression from 15 patients, plotted on a logarithmic Y-axis. Delta CT values were obtained using L32 as the housekeeper gene, then converted to ddCT (2^CT^) for all fold change calculations. Student’s paired t test was used to determine significance, where ^∗∗^*p* < 0.001 and ^∗∗∗^*p* < 0.0001. Significance was achieved after adjustment for multiple testing. **(B)** Assessment of SDC1 and SDC4 protein expression in HMD vs. LMD tissues. Representative IHC images are displayed with intensity data (% DAB positivity per tissue area) plotted underneath. Lobular epithelium, *n* = 4 pairs for SDC1, *n* = 9 pairs for SDC4; **(C)** Stromal regions, *n* = 35 pairs for SDC1, *n* = 30 for SDC4. **(D)** Ductal vs. lobular epithelium (HMD only shown) for SDC1 or SDC4, *n* = 8 pairs for SDC1 and *n* = 12 for SDC4. **(E)** Ductal vs. lobular epithelium (HMD only shown) for HPSE, *n* = 12 pairs analyzed. Student’s paired *T*-test was used to determine significance, where *p* < 0.001 is indicated by ^∗∗^ and *p* < 0.0001 is indicated by ^∗∗∗^. All images were captured at 10x magnification, scale bar = 50 μM.

Cut sections of HMD vs. LMD, were fixed overnight in 4% paraformaldehyde made up in PBS at 4 degrees, processed to paraffin, sectioned and stained with various primary antibodies (detailed in Table 3) on an automated system (Ventana Discovery Ultra, Roche, Switzerland). “Intensity” (as specified on Y axes in Figures 1A–E) represents per cent DAB positivity per tissue area, and was determined using ImageJ, where DAB brown positive tissue regions were separated using a Color Deconvolution plugin (H DAB), a threshold applied, and percentage positive area quantified.

**TABLE 3:** Primary antibodies used in this study for Immunohistochemistry.

Antigen	Antibody	Dilution	Supplier
SDC1	Monoclonal Rabbit Anti-Human	1:100	Abcam (ab130405)
SDC4	Polyclonal Rabbit Anti-Human	1:100	Abcam (ab24511)
TFF1	Polyclonal Rabbit Anti-human	1:1000	Invitrogen (PA5-31863)
HPSE (Hpa)	Monoclonal Mouse Anti-Human	1:100	ThermoFisher Scientific (MA5-16130)

### Patient-Derived Explants of Normal Breast Tissue

Briefly, ∼2 cm^3^ sized pieces of breast tissue excised from high, medium or low MD regions, determined from a slice mammogram by a radiologist (TL), were cut into smaller pieces of ∼0.5 cm^3^ in size and placed onto a gelatin sponge scaffold submerged in media inside a well of a 24-well culture plate. Viability of this tissue was then maintained for up to 2 weeks in 24 well plates, submerged in basal media as previously described (Centenera et al., 2018) to sustain tissue viability and hormone responsiveness. For HPSE inhibitor treatment, this basal media comprised of RPMI containing 10% FBS (Thermofisher Scientific, Australia), 1 x penicillin and streptomycin (Merck, Australia), 100 μg/mL hydrocortisone (Merck, Australia) and 100 μg/mL insulin (Merck, Australia) was supplemented either one of the following: 100 μM fondaparinux (sourced from Princess Alexandra Hospital Pharmacy), 100 μM PI-88, 10 μM PG545 control or 10 μM PG545 (provided by V. Ferro). For estrogen (1nM Estradiol, Merck, Australia) and tamoxifen (1 μM 4-hydroxy-tamoxifen, Merck, Australia) treatments, phenol red-free RPMI was supplemented with 10% charcoal-stripped FBS (Thermofisher Scientific, Australia), with all other supplements the same as for the HI experiments. For all treatments, media within 24 well plates was replenished twice weekly.

### NMR Measurements

The individual breast explants were removed from the underlying sponge and placed in a well within a dry 24 well plate for Portable NMR measurements. For PG545 and E2 treatments, NMR T1 values of the samples were measured using saturation-recovery pulse sequence on a PM5 NMR-MOUSE instrument (Magritek, Wellington, New Zealand) as previously described (Tourell et al., 2018; Huang et al., 2019). All saturation-recovery curves exhibited mono-exponential recovery. The curves were least-squares fitted with a three-parameter exponential fitting function, and the time constants of the recovery were taken as the respective T1 values. For SSTN treated explants,% water was measured using two approaches: (1) Carr-Purcell-Meiboom-Gill (CPMG) decay followed by an inverse Laplace transform, as described in Ali et al. (2019). This approach yielded T2 relaxation spectra with two peaks (Fat and Water), whose relative areas were taken as the content of the respective chemical component within the tissue; (2) Diffusion NMR measurements followed by two-component least-squares fitting of the diffusion attenuation plot (Huang et al., 2019). The amplitudes of the two components of the fit (Fat and Water) were taken as the content of the respective chemical component within the tissue.

### Picrosirius Red (PSR) Staining and Analysis Under Polarized Light

PSR staining of human breast tissues to detect fibrillar collagen (collagen I) was performed as previously described (Kiraly et al., 1997). Quantification of staining was performed using specific ImageJ macros written to identify and quantify red color in PSR-stained sections illuminated with polarized light to visualize thick fibrillar collagens.

### ELISA for Shed SDC1 and SDC4 in Explant Media

SDC1 and SDC4 proteins shed into the conditioned media surrounding explanted normal mammary tissue at experimental endpoint (day 14) were quantified using the SDC1 and SDC4 ELISA kits (Raybiotech, United States) according to the manufacturer’s instructions, following dilution of the conditioned media 1:4 with the kit dilution buffer.

### Statistics

Pearson’s *r* coefficients and respective *p*-values and the Student’s *T*-test (used to assess the differences in MD changes and gene expression levels between the different treatment groups) were calculated using GraphPad Prism 8.

## Results

### HMD vs. LMD Pairwise ECM Gene and Protein Expression Analysis

#### RT-qPCR Data

As shown in Figure 1, RT-qPCR assessment of a range of HSPGs showed that mRNA levels of SDC1 and SDC4 were significantly more abundant, 14 and 7-fold higher, respectively, in HMD vs. LMD paired tissue from 15 women. In contrast, levels of GPC3 and GPC6 were significantly decreased by 0.8- and 0.65-fold, respectively. Significance was achieved after adjustment for multiple testing. The proteins coded by these mRNAs are substrates for HPSE. We did not find any difference in HPSE expression between HMD and LMD. Given that increased stroma is characteristic of HMD (Huo et al., 2015), postulated in this study to be driven by HSPG abundance, SDC1 and SDC4 were thus taken forward for further analysis.

#### Localization of SDC1 and SDC4 in Normal Mammary Tissue

Stromal, not epithelial, localization of SDC1 protein has been implicated in promoting breast density and breast cancer (Lundstrom et al., 2006). Therefore, we performed a detailed IHC investigation of SDC1 and SDC4 PGs in epithelial and stromal compartments of paired HMD vs. LMD tissues (Figures 1B–E). No significant difference was observed for epithelial SDC1 or SDC4 protein expression in HMD vs. LMD (Figure 1B), however SDC1 expression was higher in stromal regions derived from HMD compared with LMD (Figure 1C). This was not observed for SDC4, suggesting SDC1 abundance may drive HMD. Both SDC1 and SDC4 protein levels appeared more abundant in ductal rather than in lobular epithelia, irrespective of MD status (Figure 1D). No difference in HMD vs. LMD epithelial vs. stromal regions were observed for HPSE *(data not shown)*, however HPSE displayed the same expression pattern as for SDC1 and SDC4 in that it was higher in ductal vs. lobular epithelium (Figure 1E).

### Establishment of a Patient-Derived Explant Model to Assess MD Change

**FIGURE 2:**
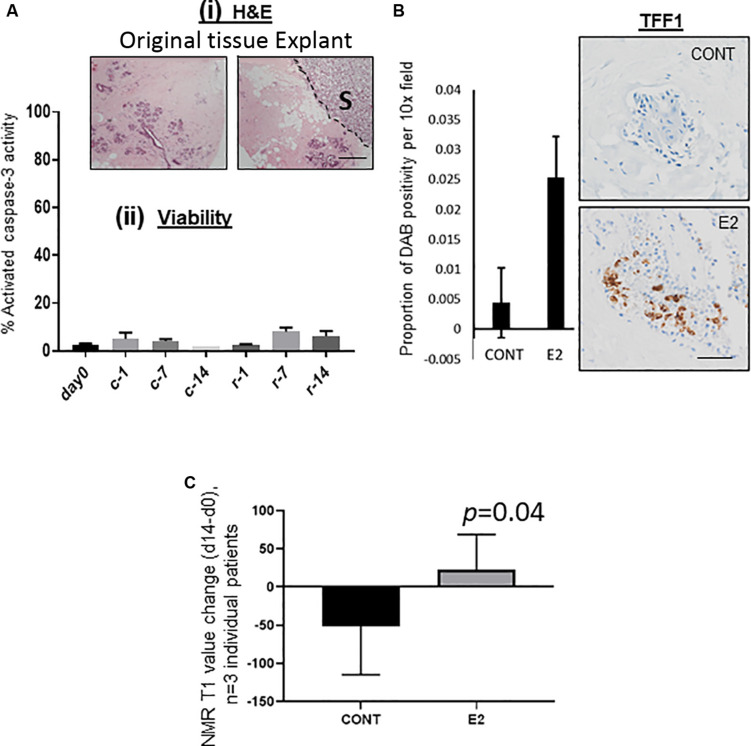
**(A)** (i) Morphological comparison of original tissue at *t* = 0 and following 14 days as explant culture. S denotes position of the sponge, delineated from the explant tissue by a dotted line. (ii) Viability of explanted tissue up to 14 days as assessed by% activated caspase 3 activity as measured by IHC. C, clear media (Phenol-red free); r, red media (Phenol-red containing). **(B)** 1nM Estradiol treatment of PDEs led to a clear upregulation of the E2-signaling pathway protein TFF-1, images and quantification are typical representative data of 3 individual patients. **(C)** Mammographic density change (d14–d0) in E2 treated mammary tissue explants as measured by the change in NMR T1 values. For part **(C)** only, Student’s paired *T*-test was used to determine significance, with *p*-values displayed on graph, *n* = 3 individual patient tissue tested. For all images shown, 10x magnification was used, scale bar = 50 μM.

All published reports that have examined MD change have been longitudinal and in human population cohorts (Junkermann et al., 2005; Cuzick et al., 2011; Jacobsen et al., 2017). We sought a means to test the effect of exogenous agents on MD in a culture setting using intact mammary tissue, and adopted a whole-tissue explant model (patient-derived explant, PDE), originally designed for the study of human cancers *ex vivo* (Centenera et al., 2018). These original studies describe the culture of cancer tissue for periods of time measured in days, however we hypothesized that to observe any measurable change in density in normal tissue, treatment times would need to be extended to weeks. Initial concern in adopting this extended treatment time was that tissue viability would be compromised. However, as shown in Figure 2Ai, morphology of the PDEs after 2 weeks was comparable to tissue at day 0, and we found minimal activated caspase-3 activity in medium MD explant tissue cultured for 2 weeks, indicating that apoptosis was not a major event in these tissues during this time (Figure 2Aii).

#### Increase in MD With Estradiol Treatment of PDEs

We then treated our medium MD PDEs with 1nM estradiol for 14 days, as estrogen is an implicated driver of MD (Greendale et al., 1999; Byrne et al., 2017). After 14 days, we found the estrogen receptor signaling pathway mediator TFF-1 protein to be upregulated in comparison to the control (Figure 2B).

We have demonstrated that NMR T1 (and to a lesser extent, T2) values correlate strongly with microCT-derived% HMD in *ex vivo* explants, such that T1 values may be used as a surrogate marker for MD (Huang et al., 2019). We therefore chose to measure T1 as a surrogate marker of MD in our PDE model at *t* = 0 and *t* = 14, to determine MD change. As shown in Figure 2C, in all three experiments in which tissue from women was tested, estrogen increased MD change (d14–d0) compared to control treated (*p* = 0.04). In another patient, tamoxifen negated estradiol-mediated MD increases over the experimental period (14 days, Supplementary Figure S1).

#### Coupling of HPSE With SDC1 and SDC4 Induction With ERα Pathway Activation

**FIGURE 3:**
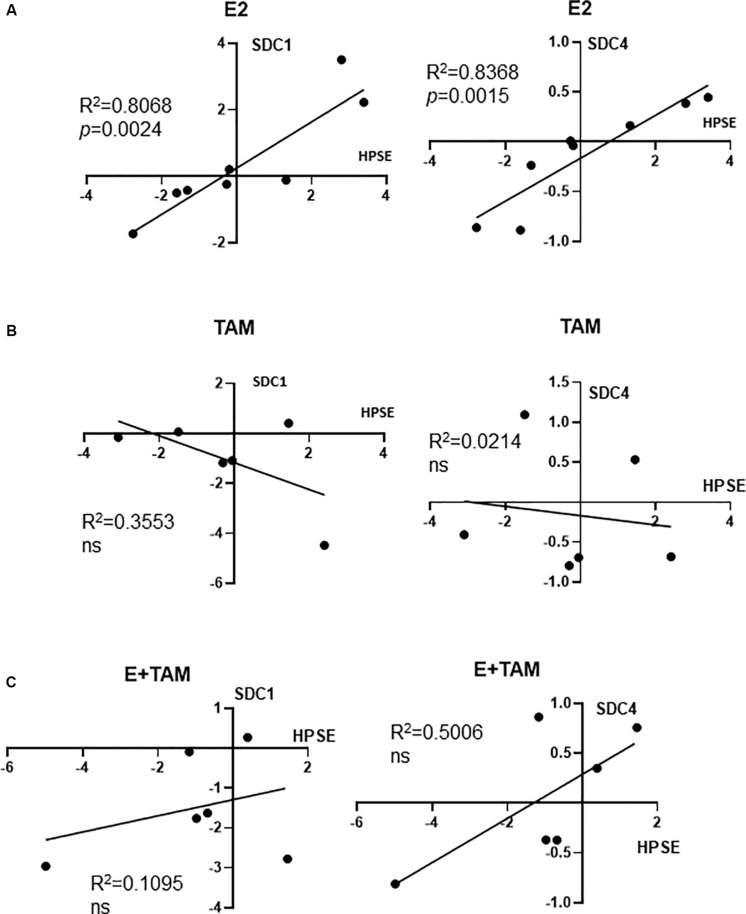
RT-qPCR data from hormone treated explants: Correlation of HPSE with SDC1 or SDC4 in E2 **(A)**, Tamoxifen **(B)**, and E2 + Tamoxifen **(C)** treated explants. Expression data shown is normalized to control-treated explant mRNA control (ddCT). Each data point represents the average expression from 3 explants from 1 patient, where *n* = 8 for part **(A)**, *n* = 6 for parts **(B,C)**. Pearson’s *r* coefficient is shown and where *p*-values stated where *p* < 0.05; ns, not significant.

Since it is known that estrogen upregulates HPSE mRNA (Elkin et al., 2003), that HPSE is a key modulator of both SDC1 and SDC4 abundance in the breast reviewed in Sarrazin et al. (2011), and that cleavage of SDC1 leads to SDC1 mRNA induction (Ramani et al., 2012), we investigated whether the E2-HPSE-SDC1 axis could be relevant in our human mammary tissue explants. As shown in Figure 3A, significant correlations were observed for both SDC1 and SDC4 with HPSE in E2-treated explants. Each of the data points shown in this Figure represent the average of 3 explants from 1 patient (independent biological replicates), and in Figure 3A, data from 8 individual patients was analyzed. The correlation observed in Figure 3A was lost when tissue explants were either treated with tamoxifen alone (Figure 3B) or co-treated with E2 and tamoxifen (Figure 3C), where 6 individual patient’s explant tissue was examined. Furthermore, HPSE protein was found to be more abundant in E2-treated PDEs (Supplementary Figure S2A). These findings suggest a coupling of HPSE with SDC1 and SDC4 induction where the ERα pathway was activated, however, further studies with larger numbers are required to confirm this, including a more thorough examination of *in vitro* and *in vivo* tissues.

### Effect of HPSE Inhibition in PDEs

Given that we were able to detect an increase in MD in the PDE model with estradiol, and that HPSE regulation of SDC may be implicated in mediating this change, we decided to use the PDE model to inhibit HPSE specifically and assess the effect on MD and gene expression outputs.

#### HPSE Inhibition Reduced MD

**FIGURE 4:**
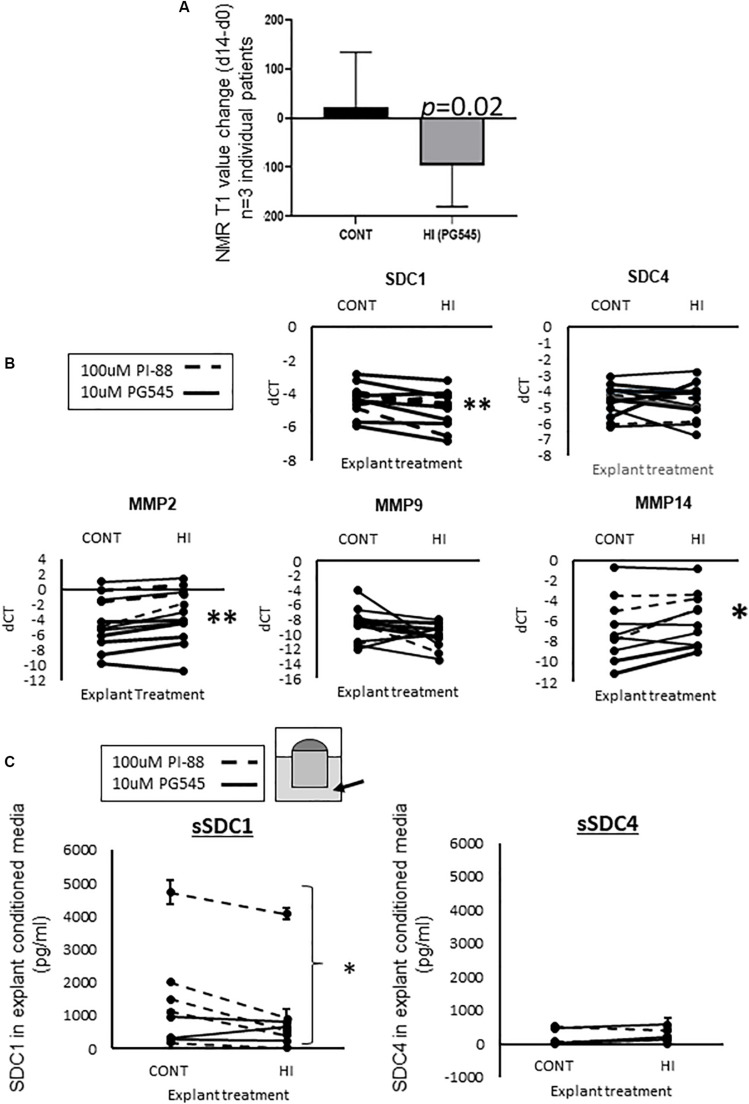
**(A)** MD change (d14–d0) in PG545 treated mammary tissue explants as measured by the change in NMR T1 values, Student’s paired *T*-test was used to determine significance, with *p*-values displayed on graph, *n* = 3 individual patient tissue tested. **(B)** RT-qPCR data from HI treated explants. Dotted line denotes 100 μM PI-88 treated explants, where control is 100 μM Fondaparinux; solid line denotes 10 μM PG545 treatment, where control is media alone. More gene expression data is found in Supplementary Figure S3. Delta CT values are plotted, determined using using L32 as the housekeeper gene. **(C)** Inhibition of HPSE led to a decrease in the abundance of shed SDC1 in explant conditioned media (Student’s paired *T*-test used to determine significance, ^∗^denotes *p* < 0.05). Dotted line: PI-88, solid line: PG545. For SDC1, *n* = 8 pairs, for SDC4, *n* = 6.

We examined the effect of the HS mimetic PG545 (Chhabra and Ferro, 2020) in the PDEs for the same period of time as for estradiol (14 days). IHC assessment of HPSE revealed that it was predominantly located around glandular epithelia, and 10 μM PG545 led to the strongest proportional reduction in peri-glandular HPSE protein content (Supplementary Figure S1A). Therefore, 10 μM was used in further studies. As shown in Figure 4A, in all three experiments in which breast tissue from women was tested, PG545 decreased MD relative to the respective control, as indicated by NMR T1 change (d14–d0, *p* = 0.02).

#### Gene Expression Changes Following HPSE Inhibitor Treatment in PDEs

Analysis of the effects of HPSE inhibition on various HSPG mRNA levels in explanted tissue at endpoint (14d) by RT-qPCR revealed a significant reduction in SDC1 expression, but not in SDC4 or other HSPGs (Figure 4B and Supplementary Figure S3), again indicating a complex transcriptional interplay between HSPG and SDC1. Given that HPSE activity has also been shown to positively influence MMP14 expression (Gomes et al., 2015) and is associated with MMP9 (Abu El-Asrar et al., 2016) we also examined MMP expression in the control vs. treated tissues. Of the MMPs that displayed expression above baseline (MMP-2, -9 and -14), MMP2 and MMP14 showed a significant increase after HPSE inhibitor (HI) treatment (Figure 4B).

#### Effect of HPSE Inhibitor Treatment on Shed SDC1 and SDC4

The abundance of SDC1 or SDC4 protein was examined in the conditioned media of PDEs at *t* = 14, as an indicator of HPSE activity, given that the form of the protein detected by this means has been shed from the cell membrane (Yang et al., 2007).

As shown in Figure 4C, there was, on average, 2 to 8-fold more SDC1 than SDC4 protein shed into the media, and only SDC1 displayed a significant reduction in abundance with HPSE inhibition (*p* < 0.05).

### SDC1-Specific Inhibition Using SSTN Reduced Fibrillar Collagen and MD

Given that gene expression and shedding for SDC1 (but not SDC4) was significantly reduced with heparanase inhibition, we hypothesized that SDC1 was mediating the observed MD change shown in Figure 4A. But how could SDC1 mediate MD? McConnell and colleagues found that peri-ductally aligned collagen is correlated with MD (McConnell et al., 2016), and a direct role for membrane-bound SDC1 in physically aligning collagen fibers has been described (Yang and Friedl, 2016). We therefore decided to interrogate the connection between SDC1 and collagen and MD in our PDE model.

SSTN is a peptide designed and validated to block SDC1-binding to ITGαvβ3, and hence ITGαvβ3 binding to peri-ductal collagen, but also with Insulin-like growth factor 1 receptor (IGF1R) (Rapraeger, 2013). First, we validated its effectiveness in our own hands to block MDA-MB-231 breast cancer cell binding to vitronectin, an interaction that specifically requires SDC1-ITGαvβ3 complexes. As shown in Supplementary Figure S4, 30 μM of SSTN reduced MDA-MB-231 cell binding and this concentration was therefore carried forward into our PDE experiments.

**FIGURE 5:**
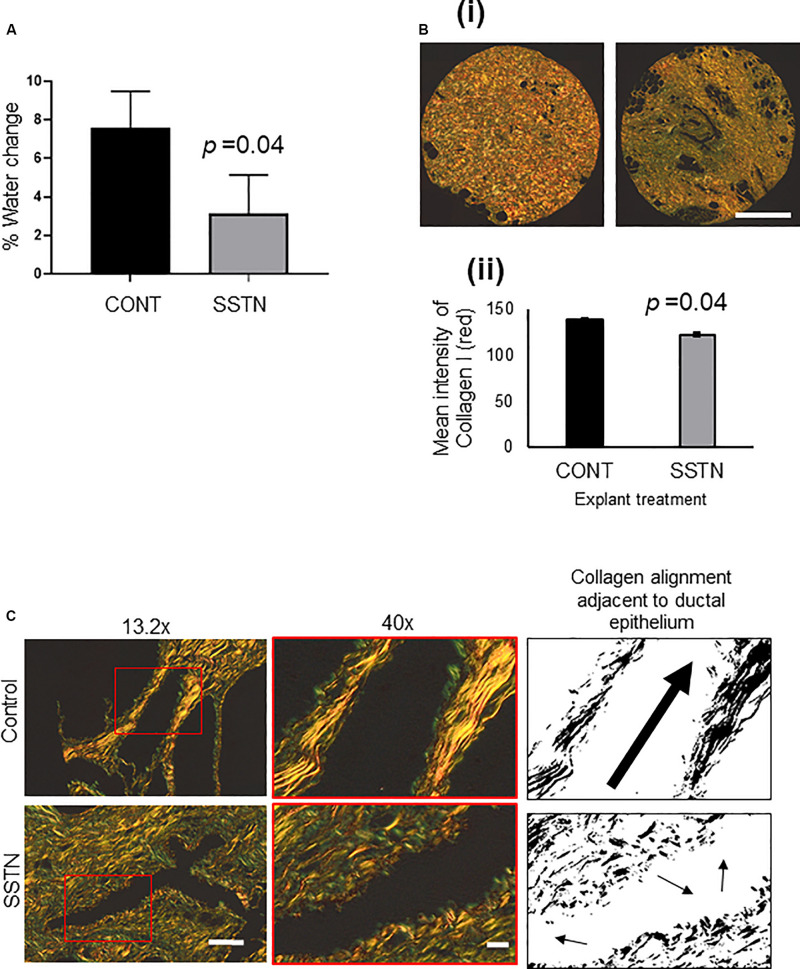
**(A)** MD change (d14–d0) in SSTN treated mammary tissue explants as measured by the change in % water diffusion values as determined by single-sided NMR. **(B)** (i) Picrosirius red staining of PDE tissue microarray and imaging with polarized light to visualize dense collagen fibers; (ii) quantification of dense collagen represented as the mean intensity of red fibers visualized by polarized light (data from *n* = 3 patients, 3 explants quantified per patient). Results are the mean and error bars represent standard error. *P*-value was determined using Student’s paired *T*-test. Images captured at 4x magnification, scale bar = 200 mm. **(C)** Picrosirius red images, enlarged region of image on left is within red box, then black and white thresholded image to highlight changes to alignment of collagen adjacent to ductal epithelium after treatment with Synstatin. Images captured at 13.2 × magnification, scale bar = 100 μM, 40 × magnification, scale bar = 20 μM.

As shown in Figure 5A, SSTN significantly decreased MD relative to the respective control, as indicated by% water change determined by NMR (d14–d0, *n* = 3 individual patients, *p* = 0.02). PSR staining enhances the natural birefringence of collagen bundles. When visualized under polarized light, PSR-stained collagen I appears red while collagen III appears green, and thus PSR staining can be used to determine collagen I/III content (Vogel et al., 2015). SSTN led to a visible reduction in dense collagen, or fibrillar collagen content, as measured by PSR visualized under polarized light (Figure 5Bi,ii). Upon closer inspection, the arrangement of collagen adjacent to ductal epithelium in SSTN treated explants exhibited reduced alignment compared to the control (Figure 5C).

## Discussion

We show for the first time that MD change can be measured *ex vivo.* This significant finding has enabled a dissection of molecules involved in the maintenance of MD. Our studies illustrate a key role for HPSE in maintaining MD, with SDC1 and possibly SDC4 implicated in this process. We have previously shown that stroma is the key differentiator between high vs. low MD (Huo et al., 2015), and we show in the current study that SDC1 and -4 are both more abundant in HMD vs. LMD (Figure 1A), and that SDC1, but not SDC-4, was more abundant in the HMD stroma (Figure 1C). Although the E2-HPSE-SDC induction pathway induced SDC1 and 4 in a relatively equal manner (Figure 3A), in HPSE inhibitor-treated explant tissue, SDC1, but not SDC4, gene expression was significantly reduced (Figure 4B), and shedding of SDC1, but not SDC4, was reduced (Figure 4C). Finally, our results indicate that fibrillar collagen is important in the maintenance of MD, and that SDC1 plays a direct role in mediating this effect, as SSTN reduced MD and fibrillar collagen abundance (Figure 5).

Despite both SDC1 and SDC4 mRNA being more abundant in HMD vs. LMD (Figure 1A), HPSE modulation had a more significant, and hence functional effect on SDC1 rather than SDC4. Interestingly, SDC1 (not SDC4) is implicated in both MD and BC (reviewed in Shawky et al. (2015) where SDC1 is informative as to BC staging (Cui et al., 2017). Our work highlights that SDC1 may be a key molecular target in efforts to reduce mammographic density as a means to reduce the associated BC risk.

Shed SDC1 has a number of potential effects in promoting tumorigenesis: growth factors bound to the extracellular domain can be carried into the nucleus and potentiate proliferation (Stewart et al., 2015) and also increase wnt signaling (Alexander et al., 2000). HPSE induces SDC4 shedding, as we observed in the conditioned medium of our PDEs (Figure 4C), however the pathological significance of SDC4 shedding is in cardiac disease (Strand et al., 2015). Given that shed SDC1 in the serum is a prognostic factor in several cancers (Joensuu et al., 2002; Vassilakopoulos et al., 2005; Szarvas et al., 2016) it is likely that there are several other pro-tumorigenic effects of shed SDC1 into the microenvironment that are yet to be discovered. SDC1 shedding itself increases SDC1 expression in the cell (Ramani et al., 2012), but may also increase SDC1 expression in neighboring cells, such as fibroblasts in the stroma, a mechanism through which stromal SDC1, particularly in HMD, may be potentiated, as we have observed (Figure 1C).

We found that SDC1, SDC4, and HPSE were significantly more abundant in ductal epithelium compared with lobular epithelium (Figures 1D,E). The basis of this expression pattern is not clear, however, it may be related to differing mechanical pressure placed on ductal vs. lobular epithelia. McConnell and colleagues (McConnell et al., 2016) found significant correlations between MD and localized PSR enhanced collagen birefringence located around breast ducts (*R*^2^ = 0.86, *p* < 0.0001), but not around lobules or within distal stromal regions (*R*^2^ = 0.1, *p* = 0.33; *R*^2^ = 0.24, *p* = 0.14, respectively). The presence of this restrictive collagen lining may create a greater force on the cells underlying it. In the same way that increased load in cartilage due to exercise leads to an increase in proteoglycan content (Bird et al., 2000), perhaps this force is responsible for the observed SDC1 and SDC4 expression patterns. Indeed, we see the expression of all syndecan family members increase in human mammary epithelial cells cultured on stiff vs. soft 2D surfaces (6 kPa vs. 400 Pa, *unpublished observations*).

We observed an increase in MMP2 and MMP14 in HPSE inhibitor treated explants (Figure 4B). Similarly, HPSE knockout mice have been reported to display an upregulation of MMP2 and MMP14, where these metalloproteinases are thought to compensate for the loss of HPSE (Zcharia et al., 2009). Although decreased, SDC1 shedding was not eliminated in HPSE inhibitor-treated explants (Figure 4C). In light of the Zcharia study (Zcharia et al., 2009), this suggests that some SDC1 shedding was possible in the HPSE inhibitor-treated explants due to a MMP2 and -14 compensatory mechanism.

This study has demonstrated that the HPSE inhibitors PG545 and PI-88 are useful to interrogate the mechanistic effects of HPSE in tissue samples on MD. However, these inhibitors have undesirable anti-angiogenic and other off-target effects when used systemically (Chhabra and Ferro, 2020) and thus are unlikely MD-reducing agents to take forward to clinical trials. Tamoxifen reduces MD (Shawky et al., 2017), however, its long-term use is associated with a wide range of potential side effects, some of which are intolerable (Thorneloe et al., 2020). Both of these approaches are therefore potentially not feasible for MD reduction, although their mechanism of action provides important insight into how MD is governed, as our work alludes to, via the action of HPSE. We have uncovered a more targeted approach to MD reduction, discovered by teasing apart the connection between SDC1 and collagen. We believe this work will pave the way forward for other kinds of targeted approaches to be developed with aim to reduce MD and thus breast cancer risk.

## Data Availability Statement

All datasets presented in this study are included in the article/Supplementary Material.

## Ethics Statement

The studies involving human participants were reviewed and approved by the Metro South Hospital and Health Services, Queensland (HREC/16/QPAH/107). The patients/participants provided their written informed consent to participate in this study.

## Author Contributions

HH and ET equally conceived the study design and managed its progress. XH, GR, and HH obtained human breast tissue from respective Pathology Departments of the Princess Alexandra Hospital and Mater Private Hospital and set up human breast tissue explants for 2 week culture. XH and HH maintained explant cultures, performed gene expression and together with GR, performed IHC intensity quantification. HH performed ELISA for SDC1 and SDC4. XH and KM obtained NMR T1 measurements and KM performed analyses on this data. CS provided advice on estradiol concentrations and signaling outputs. RO performed RT-qPCR data for Figure 1A. TB provided input as to the correct statistical method to use. TL assessed breast tissue slice mammograms, and determined valid areas of HMD vs. LMD for excision. WT and TH provided hormonal reagents and technical advice on explant culture. LH provided HSPG primers and antibodies and advice in IHC interpretation. VF provided HPSE inhibitors used in explant culture, and advice in concentration optimization. All authors reviewed the manuscript prior to submission.

## Conflict of Interest

The authors declare that the research was conducted in the absence of any commercial or financial relationships that could be construed as a potential conflict of interest.

## Supplementary Material

FIGURE S1 **(A)** Dilution series (0–20 μM) of the heparan sulfate mimetic (heparanase inhibitor) PG545 and its effect on HPSE protein abundance, after 14 days treatment, as determined by IHC. Magnification 10x, scale bar = 50 μM. (B) Percentage water diffusion change data from 1 patient showing that the combination of tamoxifen with estrogen ameliorated MD increase over the treatment period (14 days).

FIGURE S2 HPSE protein abundance as measured by IHC in A. PI-88 (HPSE inhibitor) and B. E2 treated explants.% DAB positivity was quantified from at least 5 10x microscope fields. Magnification 10x, scale bar = 50 μM.

FIGURE S3 **(A)** RT-qPCR data derived from HI treated explants for the various other HSPG family members where no change in expression was observed.

FIGURE S4 MDA MB 231 adherence to two-dimensional, Vitronectin coated substrates in the presence of increasing concentration of the SDC1 inhibitor SSTN. **(A)** Cellular morphology and **(B)** Percentage adherence calculations from images shown in **(A)**. Magnification 20x, scale bar = 50 μM.
