# Corrigendum: Heparanase Promotes Syndecan-1 Expression to Mediate Fibrillar Collagen and Mammographic Density in Human Breast Tissue Cultured *ex vivo*

**DOI:** 10.3389/fcell.2021.678589

**Published:** 2021-03-31

**Authors:** Xuan Huang, Gina Reye, Konstantin I. Momot, Tony Blick, Thomas Lloyd, Wayne D. Tilley, Theresa E. Hickey, Cameron E. Snell, Rachel K. Okolicsanyi, Larisa M. Haupt, Vito Ferro, Erik W. Thompson, Honor J. Hugo

**Affiliations:** ^1^Institute of Health and Biomedical Innovation, Queensland University of Technology, Kelvin Grove, QLD, Australia; ^2^Translational Research Institute, Woolloongabba, QLD, Australia; ^3^School of Biomedical Science, Queensland University of Technology, Brisbane, QLD, Australia; ^4^Faculty of Science and Engineering, Queensland University of Technology, Brisbane, QLD, Australia; ^5^Radiology Department, Princess Alexandra Hospital, Woolloongabba, QLD, Australia; ^6^Dame Roma Mitchell Cancer Research Laboratories, Adelaide Medical School, University of Adelaide, Adelaide, SA, Australia; ^7^Cancer Pathology Research Group, Mater Research Institute, The University of Queensland, Brisbane, QLD, Australia; ^8^Mater Pathology, Mater Hospital Brisbane, South Brisbane, QLD, Australia; ^9^Genomics Research Centre, School of Biomedical Sciences, Institute of Health and Biomedical Innovation, Queensland University of Technology, Kelvin Grove, QLD, Australia; ^10^School of Chemistry and Molecular Biosciences, The University of Queensland, Brisbane, QLD, Australia

**Keywords:** mammographic density, breast cancer, heparanase, syndecan-1, NMR

In the original article, there was an error. The last author in the authorlist is shown as Honor J. Hugo when acknowledgment needs to be made of equal last authorship for Honor J. Hugo and Erik W. Thompson.

A correction has been made to the authorship section.

Xuan Huang^1, 2, 3^, Gina Reye^1, 2, 3^, Konstantin I. Momot^1, 4^, Tony Blick^1, 2, 3^, Thomas Lloyd^5^, Wayne D. Tilley^6^, Theresa E. Hickey^6^, Cameron E. Snell^7, 8^, Rachel K. Okolicsanyi^1, 3, 9^, Larisa M. Haupt^1, 3, 9^, Vito Ferro^10^, Erik W. Thompson^1, 2, 3†^ and Honor J. Hugo^1, 2, 3†^

Note: ^†^These authors share last authorship.

The authors apologize for this error and state that this does not change the scientific conclusions of the article in any way. The original article has been updated.

