# Supplementary figures and images for: Heparanase Promotes Syndecan-1 Expression to Mediate Fibrillar Collagen and Mammographic Density in Human Breast Tissue Cultured ex vivo
Source: Front Cell Dev Biol. 2020 Jul 14;8:599. doi: 10.3389/fcell.2020.00599 (PMC7373078; doi:10.3389/fcell.2020.00599)

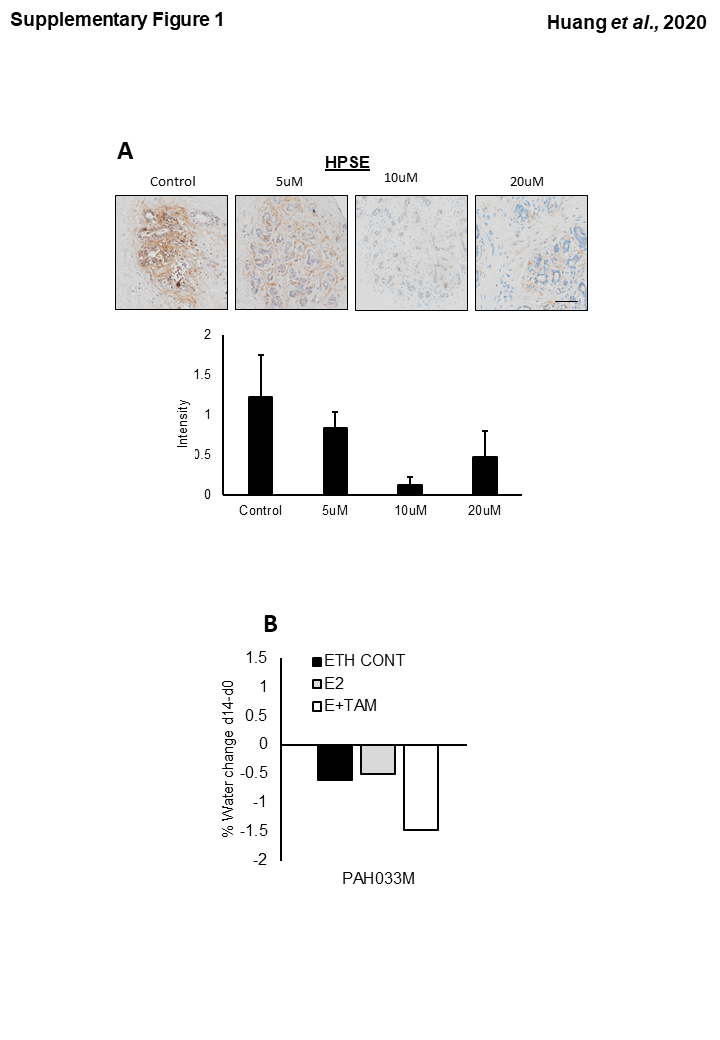

Supplement: FIGURE S1 — (A) Dilution series (0–20 μM) of the heparan sulfate mimetic (heparanase inhibitor) PG545 and its effect on HPSE protein abundance, after 14 days treatment, as determined by IHC. Magnification 10x, scale bar = 50 μM. (B) Percentage water diffusion change data from 1 patient showing that the combination of tamoxifen with estrogen ameliorated MD increase over the treatment period (14 days). [file Image_1.tif]

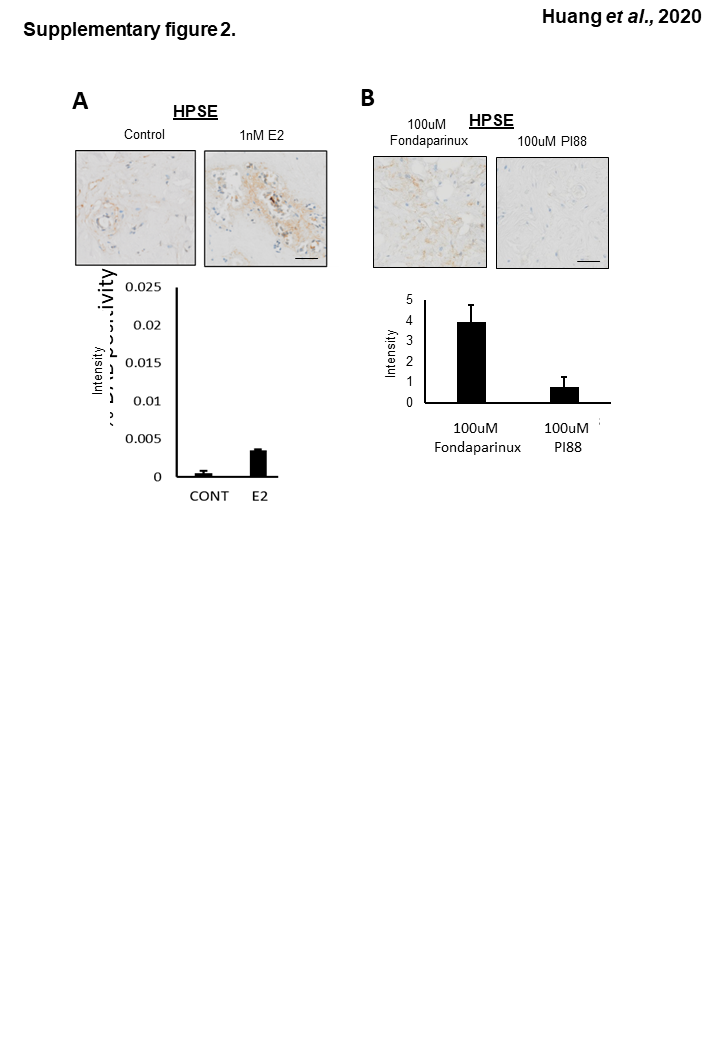

Supplement: FIGURE S2 — HPSE protein abundance as measured by IHC in A. PI-88 (HPSE inhibitor) and B. E2 treated explants.% DAB positivity was quantified from at least 5 10x microscope fields. Magnification 10x, scale bar = 50 μM. [file Image_2.tif]

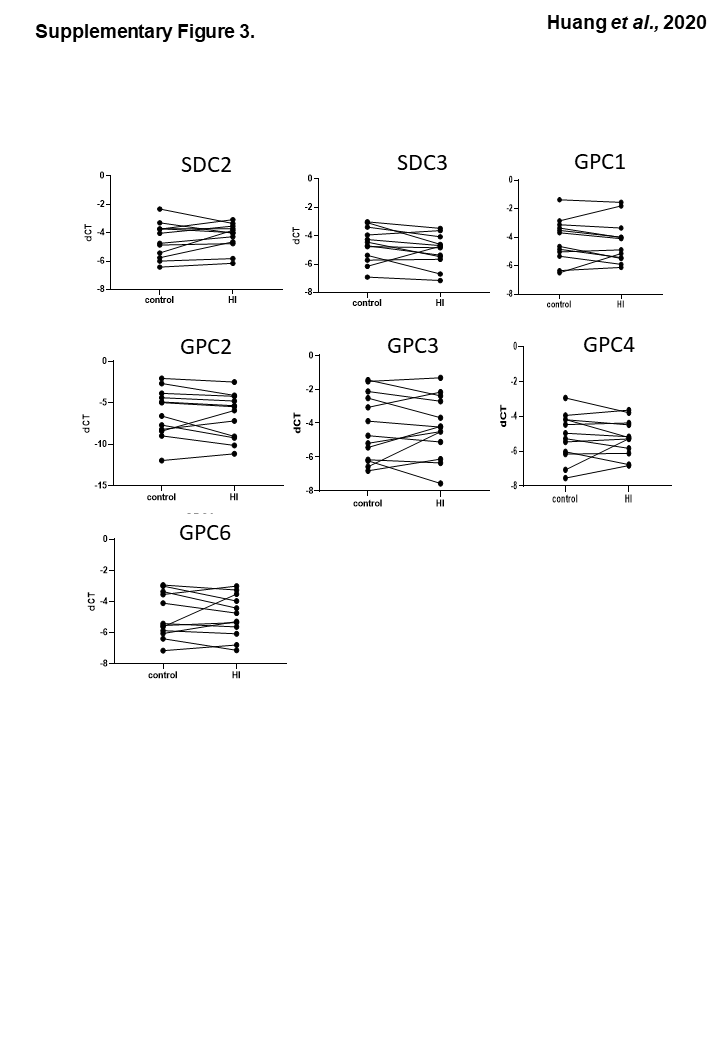

Supplement: FIGURE S3 — (A) RT-qPCR data derived from HI treated explants for the various other HSPG family members where no change in expression was observed. [file Image_3.tif]

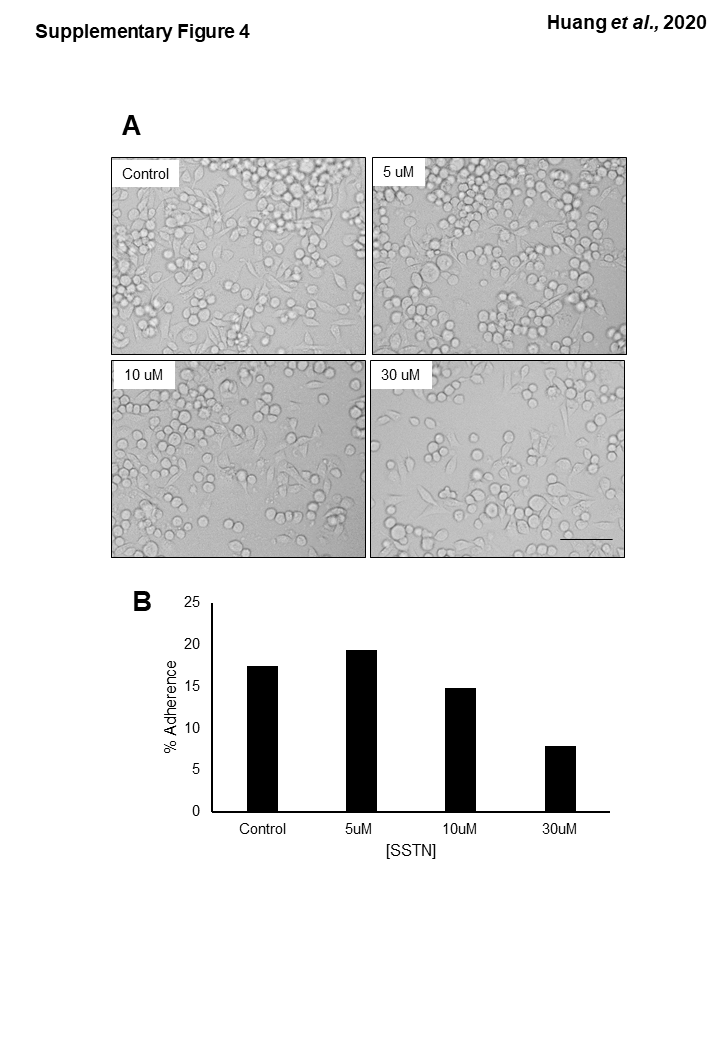

Supplement: FIGURE S4 — MDA MB 231 adherence to two-dimensional, Vitronectin coated substrates in the presence of increasing concentration of the SDC1 inhibitor SSTN. (A) Cellular morphology and (B) Percentage adherence calculations from images shown in (A). Magnification 20x, scale bar = 50 μM. [file Image_4.tif]
